# Decision-making by p53 and mTOR

**DOI:** 10.18632/aging.100166

**Published:** 2010-06-27

**Authors:** Carl G. Maki

**Affiliations:** Department of Anatomy and Cell Biology, Rush University Medical Center, Chicago, IL 60612, USA

**Keywords:** 06/16/10; accepted: 06/25/10; published on line: 06/27/10

Wild-type p53 is normally expressed at low levels and inactive due to the action of MDM2, an E3 ubiquitin ligase that binds p53 and promotes its degradation [1,2].  However, p53 is stabilized in response to various stresses, such as DNA damage or inappropriate oncogene signaling, that might otherwise predispose a normal cell toward tumorigenesis [3].  The stress-induced stabilization of p53 results from disruption of p53-MDM2 binding.  The majority of stabilized p53 accumulates in the nucleus where it functions as a transcription factor, activating expression of genes that induce either apoptosis or cell cycle arrests that can be either transient (quiescence) or permanent (senescence).  Thus, p53 eliminates cells with potentially cancer-promoting lesions by inhibiting their growth or causing them to die.  mTOR is a cytoplasmic kinase whose activity is often elevated in cancer [4].  mTOR converts signals from activated growth factor receptors into downstream events that promote cell proliferation and survival.  Previous studies have demonstrated cross-talk between the p53 and mTOR signaling pathways.  For example, p53 can activate expression of several genes, including TSC2, PTEN, IGF-BP3, and others, whose protein products can directly or indirectly inhibit mTOR activity [5].  These observations make sense given that p53 is a tumor suppressor and mTOR has more an oncogenic role in promoting cancer cell survival and proliferation.  However, more recent studies indicate that the outcome of mTOR signaling can be context-dependent.  Thus, while mTOR signaling promotes proliferation and survival under normal conditions, mTOR signaling can promote senescence under conditions in which the cell cycle is blocked [6,7].  These observations support mTOR as a hub for receipt of multiple inputs that ultimately determine cell fate.  When conditions are favorable mTOR activation promotes proliferation and survival, however, in the context of conflicting signals (e.g. growth factor signaling vs. cell cycle arrest),  the  effect  of   mTOR activation is permanent cell cycle exit (senescence).  P21 is a cyclin-cdk inhibitor, transcriptional target of p53, and potent inducer of senescence [8,9].  Blagosklonny and colleagues noted that in some cases p53 induction did not induce senescence while ectopic expression of p21 did [10].  This led to them to question the role of p53 in the senescence program, and whether p53 may actively suppress senescence.  To address this, they used a cell line in which p21 was expressed from an inducible (IPTG-driven) promoter [11].  In this cell line, transient p21 expression induced by IPTG caused the cells to undergo a senescent arrest characterized by flat-cell phenotype, expression of senescence-associated beta galactosidase, and a complete loss of proliferative potential after IPTG removal [12].  To test the effect of p53 on this senescent arrest, the authors first induced p21 by IPTG, and then induced p53 expression in the same cells by addition of Nutlin-3a, a small molecule MDM2 antagonist and potent p53 stabilizer.  Remarkably, cells in which p53 was induced by Nutlin-3a were able to resume cycling and fully recover after IPTG removal.  These results indicated that p53 expression converted the senescence response in these cells to quiescence.  The suppression of senescence they observed was associated with p53-dependent inhibition of mTOR activity [12].  In the current issue of Aging, Korotchkina et al. [13] demonstrate that shRNA-mediated knockdown of TSC2, a negative regulator of mTOR and p53 target gene [5], imposed senescence in these Nutlin-3a treated cells.  The results support a model in which p53 can suppress senescence through upregulation of TSC2 and inhibition of mTOR.

Our lab has also examined responses to p53 activation by Nutlin-3a.  Our original report demonstrated that Nutlin-3a promoted a non-permanent, tetraploid G1-arrest in two different p53 wild-type cancer cell lines (HCT116 and U2OS).  Both cell lines underwent endoreduplication after Nutlin-3a removal, giving rise to tetraploid clones resistant to therapy-induced apoptosis [14].  More recently, we demonstrated that Nutlin-3a could promote a tetraploid G1-arrest in multiple p53 wild-type cell lines [15].  However, some cell lines underwent endoreduplication to relatively high extents after Nutlin-3a removal while other cell lines did not.  The resistance to endoreduplication observed in some cell lines was associated with a prolonged 4N arrest after Nutlin-3a removal.  Knockdown of either p53 or p21 immediately after Nutlin-3a removal could drive endoreduplication in otherwise resistant 4N cells.  Finally, 4N arrested cells had diminished p53 expression, but retained high levels of p21.  Moreover, these cells expressed senescence-associated beta galactosidase, had a flattened cell phenotype, and underwent a permanent proliferation block (senescence) after Nutlin-3a removal.  These findings demonstrated that transient Nultin-3a treatment can promote senescence in 4N cells of certain cell lines associated with persistent p21 expression and resistance to endoreduplication.  In terms of a model, the results suggest p53 is required to initiate Nutlin-3a-induced senescence by increasing p21 expression, but is not required to maintain senescence.  In light of the findings by Blagosklonny and colleagues, we would speculate that the diminished level of p53 restores mTOR activity in these 4N cells, and that mTOR activity, as well as elevated p21, are required for their senescent arrest.

## Implications / Questions

The findings of Blagosklonny and colleagues have obvious clinical implications, particularly regarding the use of Nutlin-3a or other MDM2 antagonists in cancer therapy.  Nutlin-3a is in preclinical stages of develop-ment and has tremendous potential as a therapeutic agent against p53 wild-type cancers.  Indeed, Nutlin-3a treatment inhibited the growth of multiple p53 wild-type human tumor cell lines grown as xenografts in nude mice [16,17], and Nutlin-3a causes a pronounced cell cycle arrest or apoptotic response in p53 wild-type cancer cell lines [17,18].  However, if p53 activation by Nutlin-3a can suppress senescence in cancer cells and cause them to arrest in a quiescent state, then these cells could recover after treatment and resume cycling.  This would conceivably limit the effectiveness of Nutlin-3a-based therapies.  In this issue of Aging it was reported that the status of the mTOR pathway can determine, at least in part, the choice between senescence and quiescence in Nutlin-3a and p53-arrested cells [13].  In fact, Nutlin-3a failed to inhibit mTOR in melanoma-derived cell lines and mouse embryo fibroblasts that undergo senescence as their primary response to p53 activation.  The findings imply that Nutlin-3a could be effective as a single treatment agent, but only against cancers in which p53 fails to inhibit or only partially inhibits mTOR.  Studies to determine the molecular basis for why p53 can inhibit mTOR in some cell lines but not others will be closely watched.

Nutlin-3a stabilizes p53 in a non-genotoxic fashion without inducing DNA damage [19], and a question that arises is the extent to which the findings of Blagosklonny's group can be generalized to the p53 stress response.  The stabilization and activation of p53 in response to DNA damaging stress result from post-translational modifications (phosphorylations) in p53 and MDM2 that disrupt their interaction [3].  These same modifications can also influence promoter selectivity, thus directing p53 to different target genes [20,21].  DNA damage can also activate signaling pathways independent of p53 that influence transcript-tion, DNA repair, etc.  Blagosklonny and  colleagues increased p53 expression through mostly non-genotoxic mechanisms (Nutlin-3a treatment, Ad-p53 infection) and showed this p53 could suppress senescence through mTOR inhibition [6,7,12]. It remains to be seen the extent to which p53 induction in the context of a larger DNA damage response similarly suppresses senescence.  On a related note, Nutlin-3a has also been considered as a combinatorial agent for therapy with DNA damaging chemotherapeutic drugs.  Thus, it will be important to clarify the extent to which p53 induced by combination Nutlin plus DNA damaging stress suppresses senescence.

A final question is why p53 would suppress senescence and favor quiescence.  A quiescent-like arrest mediated by p53 has been well described.  In response to low levels of DNA damage p53 induces transient G1 and G2-phase arrests that allow cells time to repair their DNA [22].  Once DNA repair is complete, cells can resume entry into S-phase and mitosis.  In this context, quiescence allows the cells to recover from whatever stress is inducing p53.  Blagosklonny and colleagues showed p53 induction by Nutlin-3a suppressed senescence and favored quiescence, suggesting p53 was functioning in a way to allow stress recovery.  Again, since Nutlin-3a induces p53 in a non-genotoxic fashion, it would be interesting to know whether this is a property specific to p53 induced by non-genotoxic means or, in the case of low level DNA damage, whether it is a property of p53 molecules that have not been subject to damage-induced modifications.
